# Demonstration of an energy-efficient Ising solver composed of Ovonic threshold switch (OTS)-based nano-oscillators (OTSNOs)

**DOI:** 10.1186/s40580-024-00429-2

**Published:** 2024-05-23

**Authors:** Young Woong Lee, Seon Jeong Kim, Jaewook Kim, Sangheon Kim, Jongkil Park, YeonJoo Jeong, Gyu Weon Hwang, Seongsik Park, Bae Ho Park, Suyoun Lee

**Affiliations:** 1 Center for Neuromorphic Engineering, Korea Institute of Science and Technology, Seoul, 02792 Korea; 2 Department of Physics, Division of Quantum Phases & Devices, Konkuk University, Seoul, 05029 Korea; 3 Core Facility Center for Quantum Characterization/Analysis of Two-Dimensional Materials & Heterostructures, Konkuk University, Seoul, 05029 Korea; 4 Division of Nano & Information Technology, Korea University of Science and Technology, Daejon, 34316 Korea; 5 Dept. of Materials Science and Engineering, Korea University, Seoul, Republic of Korea; 6 Materials Science & Engineering, Seoul National University, Seoul, Republic of Korea

**Keywords:** Ising solver, Ovonic threshold switch (OTS), Oscillator-based computing

## Abstract

As there is an increasing need for an efficient solver of combinatorial optimization problems, much interest is paid to the Ising machine, which is a novel physics-driven computing system composed of coupled oscillators mimicking the dynamics of the system of coupled electronic spins. In this work, we propose an energy-efficient nano-oscillator, called OTSNO, which is composed of an Ovonic Threshold Switch (OTS) and an electrical resistor. We demonstrate that the OTSNO shows the synchronization behavior, an essential property for the realization of an Ising machine. Furthermore, we have discovered that the capacitive coupling is advantageous over the resistive coupling for the hardware implementation of an Ising solver by providing a larger margin of the variations of components. Finally, we implement an Ising machine composed of capacitively-coupled OTSNOs to demonstrate that the solution to a 14-node MaxCut problem can be obtained in 40 µs while consuming no more than 2.3 µJ of energy. Compared to a previous hardware implementation of the phase-transition nano-oscillator (PTNO)-based Ising machine, the OTSNO-based Ising machine in this work shows the performance of the increased speed by more than one order while consuming less energy by about an order.

**Supplementary Information:**

The online version contains supplementary material available at 10.1186/s40580-024-00429-2.

## Introduction

Combinatorial optimization problems (COPs) are commonly found in everyday life, for example, schedule-planning, travel-planning, resource allocation, etc., and are solved in brute-force forms or rather sophisticated algorithmic forms. As COPs are expanding their application to logistics, routing in IC (integrated circuits) design, drug discovery, etc., there is a growing demand for an energy- and time-efficient solver for COPs since conventional computers with the von Neumann architecture are not good at solving these kinds of problems [1]. A promising alternative computing method is to use the so-called “compute-by-physics” strategy, where a COP is translated into a complex physical system which, ruled by the physics same as in the problem [2–4], evolves into the ground state leading to a solution to the problem. One example is the Ising machine (IM), a system composed of spins coupled by the exchange interaction, whose ground state naturally gives a solution to the MaxCut problem [5]. In practical implementations of IM, the Ising spins can be replaced with coupled oscillators with phase being quantized at a few values by the same physics governing the Ising spins. Prototypical large-scale IMs were demonstrated by using optical parametric oscillators (OPO) [6–8], showing excellent performance of Ising solvers compared to other competitors. Nevertheless, such OPO-based IM has a drawback in their scalability because it necessitates the long fiber ring cavity requiring a lot of space, bulky optical components such as a parametric phase-sensitive amplifier, and optical tables to minimize the perturbation by vibrations. To resolve these problems, instead of using optical wave, spinwave- and surface acoustic wave-based IMs (SWIM [9] and SAWIM [10], respectively) were introduced very recently. These techniques, however, rely on the time-multiplexing technique for the coupling between oscillators, where the coupling strength is calculated in an FPGA (field-programmable gate array) and imposed to oscillators sequentially, spending most of the time in the delay line. It possibly poses a problem of the time-to-solution and the energy-to-solution increasing with the number of oscillators.

As alternative approaches, IMs based on electrical oscillators have been studied by using, for example, ring oscillators (ROSCs) [11, 12], the spin-torque nano-oscillator (STNO), and spin-Hall nano-oscillator (SHNO) based on the magnetic tunnel junction (MTJ) [13–15], and the phase-transition nano-oscillator (PTNO) based on Mott insulators [16–19]. However, due to scalability and/or energy efficiency issues with those oscillator devices, there is still a need to find a more scalable and energy-efficient alternative for implementing a practical IM.

**Fig. 1:**
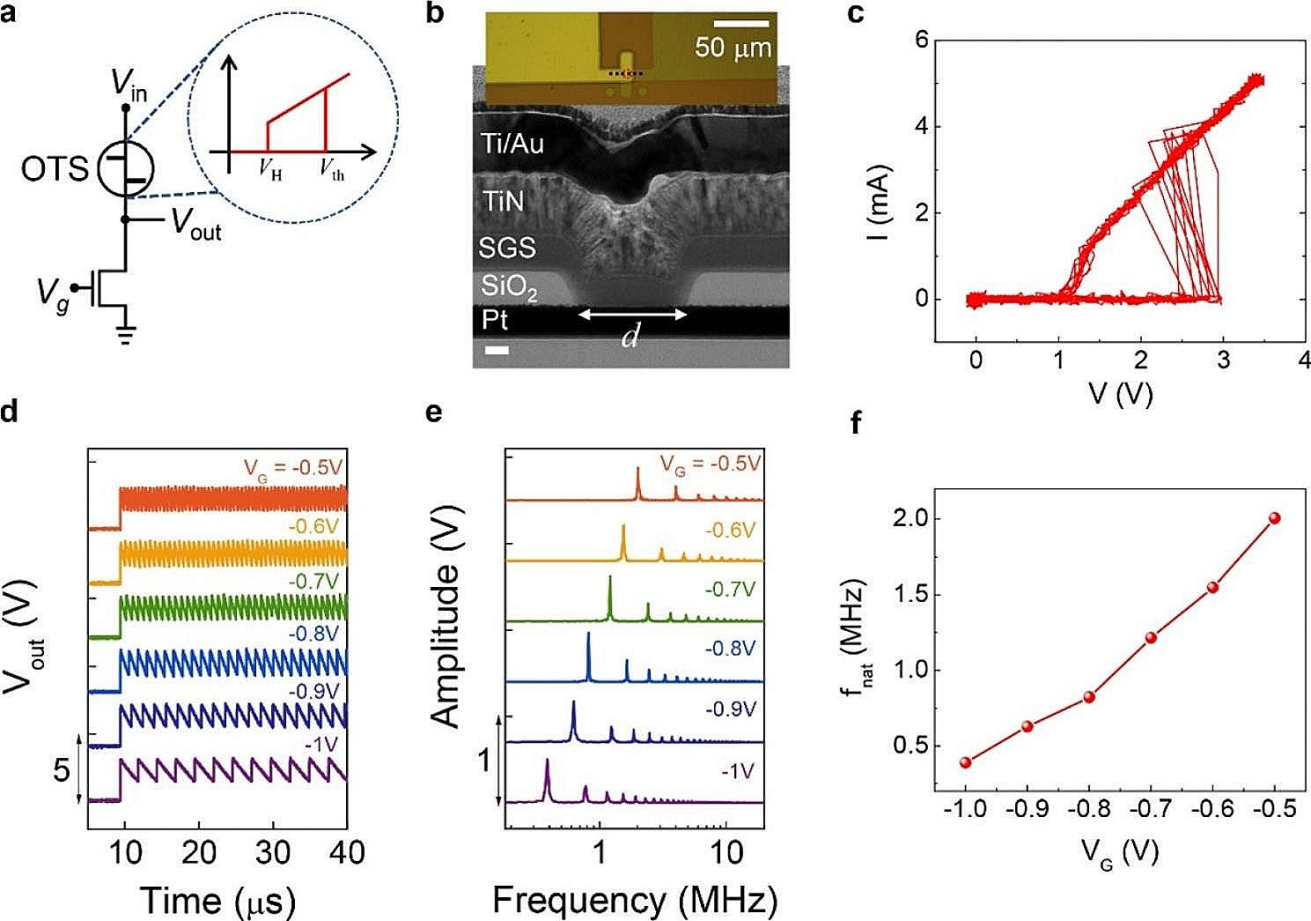
Frequency-tunable nano-oscillator based on OTS device. **(a)** 1OTS + 1FET structure as an OTS-based nano-oscillator (OTSNO). The inset shows a schematic *I*-*V* curve of an OTS device. (**b**) (top) An optical microscope (scalebar = 50 μm) and (bottom) a cross-sectional TEM image of an OTS device, where SGS means Sb_*x*_(GeSe)_1−*x*_ (*x* = 0 ∽ 0.1, scalebar = 50 nm). (**c**) Characteristic *I*-*V* curve of an OTS device (ten repetitions). (**d**) Output waveforms of the OTSNO for various *V*_G_s. (e) Fast Fourier transform (FFT)-amplitude of the output waveforms. (**f**) Dependence of the natural frequency (*f*_nat_) on *V*_G_, where *f*_nat_ is defined as the position of the primary peak in FFT

In this work, we propose a novel frequency-tunable nano-oscillator based on the Ovonic threshold switch (OTS) as a solution to this problem. This OTS nano-oscillator (OTSNO) has a structure similar to that of the PTNO with the phase-change switching device replaced by an OTS device (see Fig. 1a). Consisting of an amorphous chalcogenide sandwiched between the conducting electrodes, an OTS device shows reversible electrical switching [20], which is believed to be closely associated with the charging and discharging of the trap states inside the amorphous chalcogenide [21–29]. Such a switching mechanism endows the OTSNO with superior energy efficiency compared to the PTNO which requires Joule heating for the switching. In Fig. 1a, note that an OTS device is identified by two characteristic voltage levels, the threshold voltage (*V*_th_) and the holding voltage (*V*_H_), at which the resistance of the OTS turns low (*R*_on_) and high (*R*_off_), respectively, with a very high *R*_off_/*R*_on_ ratio (∽ 10^6^) [30–33]. For the 1OTS + 1FET structure to be an oscillator, the resistance of the FET (*R*_FET_) should have *R*_on_ < < *R*_FET_ < < *R*_off_. Under this condition, the oscillating behavior of the 1OTS + 1FET structure is easily understood considering the voltage-dividing relation between the OTS and the FET. When a voltage bias (*V*_bias_ > *V*_th_) is applied to the 1OTS + 1FET structure with the OTS in its OFF state, most of *V*_bias_ is dropped across the OTS because *R*_FET_ < < *R*_off_, turning the OTS into ON state. On the contrary, with the OTS in its ON state, most of *V*_bias_ is dropped across the FET because *R*_on_ < < *R*_FET_, turning the OTS into OFF state and completing one cycle of oscillation. Since the aforementioned charging and discharging period depends on *R*_FET_, the oscillation frequency can be controlled by the gate voltage (*V*_G_) of the FET.

For applying the OTSNO to the IM, the coupling element, mostly a capacitor and/or a resistor, should be carefully selected such that the coupled OTSNOs have an appropriate phase relationship, in-phase (IP) or anti-phase (AP). We have performed a systematic circuit simulation study to examine the phase relationship between the coupled OTSNOs depending on the strength of the coupling element and the tolerance to the variations of the oscillator devices composing the IM. Finally, by using these findings, we have constructed an IM to successfully demonstrate the solution of a MaxCut problem with 14 nodes. Finally, we present a comparison of the OTSNO with other existing nano-oscillators, showing that the OTSNO is a promising candidate for developing large-scale IMs.

## Results and discussion

Figure 1b shows an optical microscope plane-view image (top) and a cross-sectional transmission electron microscope (TEM) image of an OTS device, which has a pore-type structure with a pore size (*d*) of 300 nm. The fabrication process of the OTS device is described in detail in the Experimental Section. Figure 1c shows the characteristic current-voltage (*I*-*V*) curves of the device, which is nominally the same as Fig. 1b. In ten repeated measurements, the OTS device shows the variation in *V*_th_ within 2.5 ∽ 3.0 V while *V*_H_ is around 1 V with a negligible variation. In addition, *R*_off_ and *R*_on_ of OTS devices have been read around ∽ 10^8^ and ∽ 10^2^ Ω, respectively. Figure 1d shows *V*_out_ waveforms of a typical OTSNO with varying *V*_G_, where the OTSNO consists of an OTS device and a FET (n-MOSFET, LND150N3-G, Microchip Inc.). It is clearly shown that the natural frequency (*f*_nat_) of the OTSNO increases with *V*_G_ as expected. To quantify *f*_nat_ at each *V*_G_, the fast Fourier transform (FFT) is performed as shown in Fig. 1e and *f*_nat_, defined as the frequency at the primary peak, is plotted as a function of *V*_G_ in Fig. 1f. It is observed that *f*_nat_ is well defined and linearly proportional to *V*_G_ being adjustable in the range of 0.5 ∽ 2 MHz. These results clearly show that the oscillation in the OTSNO is composed of fundamental-frequency components and their harmonics while keeping other components negligible.

**Fig. 2:**
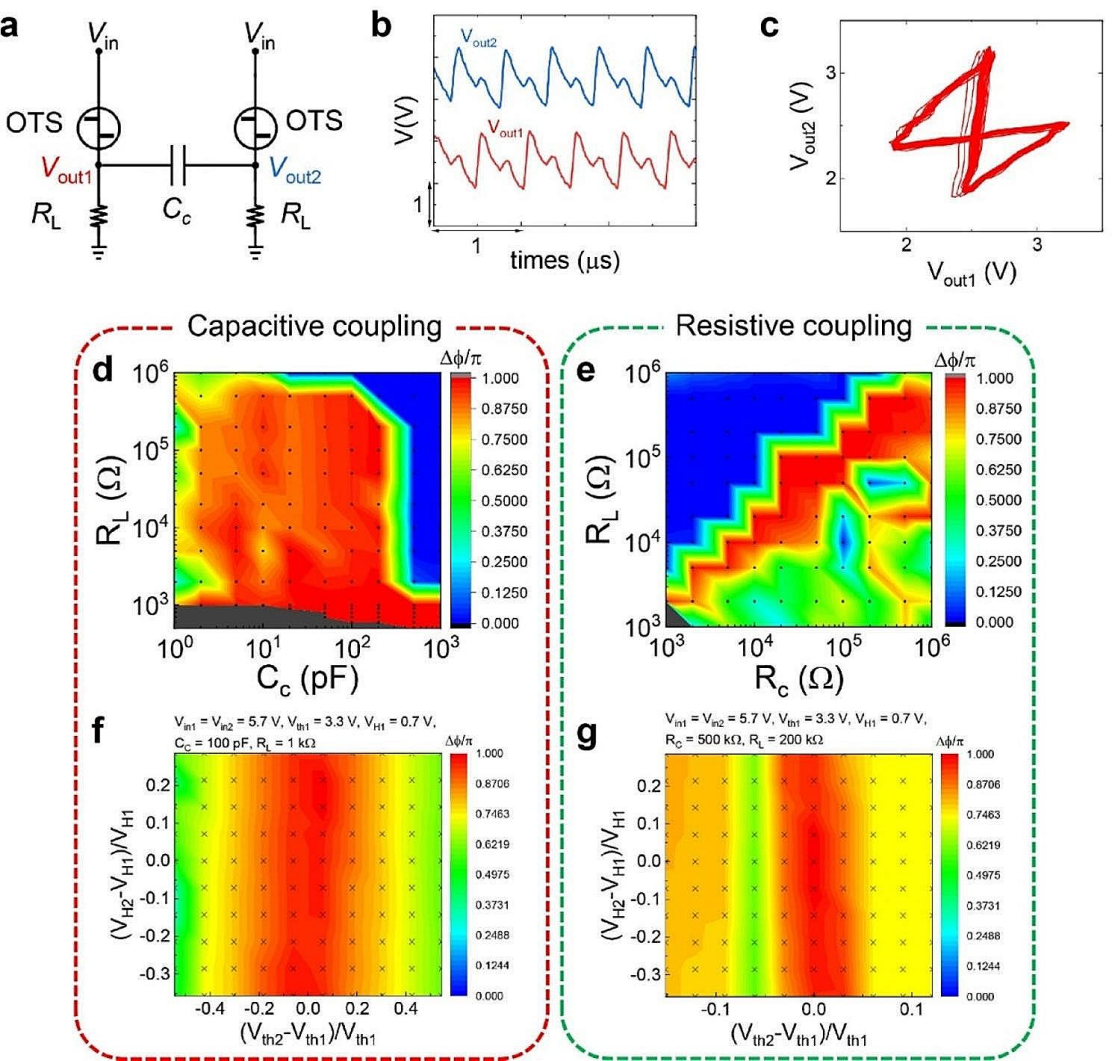
Synchronization of OTSNO. **(a**) Circuit diagram of two capacitively-coupled OTSNOs. (**b**) Output waveforms of two oscillators, where *R*_L_=2.5 kΩ and *C*_C_=100 pF, respectively. The waveforms are vertically shifted for clarity. (**c**) Phase portrait of the two coupled oscillators, which clearly shows an attractor curve resembling a butterfly indicating the anti-phase relationship between those two oscillators. (**d**), (**e**) Phase difference (*Δφ*) between two coupled oscillators as a function of the load resistance (*R*_L_) and the coupling strength (**d**) *C*_C_ and (**e**) *R*_C_, respectively). In the dark grey region, the oscillators do not show oscillating behavior. (**f**), (**g**) *Δφ* as a function of *ΔV*_th_ (= *V*_th2_ -*V*_th1_) and *ΔV*_H_ (= *V*_H2_ -*V*_H1_) for the capacitive coupling (**d**) and the resistive coupling (**e**) cases, respectively

Next, we investigate the synchronization behavior of coupled OTSNOs (see Fig. 2a). Figure 2b shows the output waveforms of two OTSNOs coupled by a capacitor. It is observed that the output waveforms of two oscillators look nearly the same with a certain degree of phase difference, demonstrating that two oscillators are synchronized to each other (for a detailed study of the synchronization behavior, see section S1 in Supplementary Information). In Fig. 2c, *V*_out2_ is plotted as a function of *V*_out1_, which presents the so-called “phase portrait” of a coupled-oscillator system. It shows a butterfly-shaped attractor curve, implying the AP relationship between the two oscillators. The attractor curve appears as a band implying the chaotic nature of the OTSNO [24, 26, 30, 34], which is desirable for the application to the IM because it helps the system to find the solution of the network configuration with the global minimum energy [35].

To examine the phase stability of two coupled oscillators, we have performed a simulation study where the circuit parameters of the system are systematically varied. Especially, we have investigated two types of coupling - capacitive and resistive coupling - with varying their values, *C*_C_ and *R*_C_, respectively. In the SPICE (Simulation Program with Integrated Circuit Emphasis) simulation, the OTS device is modeled as a voltage-controlled switch characterized by four parameters (*V*_th_, *V*_H_, *R*_on_, *R*_off_) in parallel connection with a parasitic capacitor. Figure 2d and e show the phase difference (*Δφ*) between oscillators #1 and #2 as a function of (*C*_C_, *R*_L_) and (*R*_C_, *R*_L_), respectively. Here, the parameters of both OTS devices are set to (*V*_th_, *V*_H_, *R*_on_, *R*_off_)=(3.3 V, 0.7 V, 150 Ω, 10 MΩ). *Δφ* is calculated by 2*π*(*T*_2_-*T*_1_)*f_sync_ after ∽ 200 cycles, where *T*_2_-*T*_1_ and *f*_sync_ are the time difference between adjacent peaks of oscillator #1 and #2 and the oscillation frequency in the synchronized state, respectively. It is found that the AP relationship between two oscillators is more stable in the capacitive coupling with *C*_C_ for AP relationship spanning almost two orders (2 ∽ 200 pF) independent of *R*_L_ while the range of *R*_C_ for AP relationship is relatively narrow with a strong dependence on *R*_L_.

For the practical application, an important aspect is the tolerance of the coupled oscillator system to the inevitable variation of the OTS device. Figure 2f g show *Δφ* as a function of *ΔV*_th_ (= *V*_th2_-*V*_th1_) and *ΔV*_H_ (= *V*_H2_-*V*_H1_), where we have varied *V*_th_ and *V*_H_ of OTS #2 systematically while keeping those of OTS #1 fixed. It is shown that, in the case of the capacitive coupling, the AP relationship is robust against the *V*_th_-variation of up to ± 20%, which is significantly higher compared to resistive coupling where the limit is only ± 5%. The superior stability of the AP relationship in the *C*_C_-coupled oscillator system can be explained qualitatively by the ability of the coupling capacitor to store energy. In detail, if a voltage difference is generated between two oscillators, then it can be stored in the coupling capacitor. The stored energy is returned to the oscillators making them repel each other, stabilizing the AP relationship. In contrast, since the resistor can’t store energy in the *R*_C_-coupled oscillator system, the voltage difference between oscillators generates heat dissipation in *R*_C_. This makes the oscillators lose their energy destabilizing the AP relationship in the *R*_C_-coupled oscillator system.

Another intriguing finding is that the phase relationship of the coupled OTSNOs in both cases is hardly sensitive to the variation of *V*_H_ compared to the variation of *V*_th_. It is related to the asymmetry of the output waveform (see Fig. 1d), which shows a much longer falling period compared to the rising period. Recalling the mechanism of the oscillating behavior of the OTSNO, it is easily found that the peak of the oscillation aligns with the ON-to-OFF transition of the OTS device whereas the valley point corresponds to the OFF-to-ON transition. Consequently, when the OTS reaches its peak, it transitions into a highly resistive state with *R*_off_ ∽ 10 MΩ. As mentioned above, since the OTS can be described by a parallel connection of a voltage-controlled switch and a parasitic capacitor, such a high *R*_off_ results in a large RC delay, and consequently, the elongation of the falling period compared to the rising period. During the falling period, the parasitic capacitor is charged and the voltage across it runs into *V*_th_ starting from *V*_H_. Since the charging time is mainly determined by *V*_th_, the variation in *V*_H_ has little effect on the phase relationship of the coupled OTSNOs. The output waveforms and the phase portraits at points in various regions in Fig. 2d and e are presented in the Supplementary Information (Fig. S2).

**Fig. 3:**
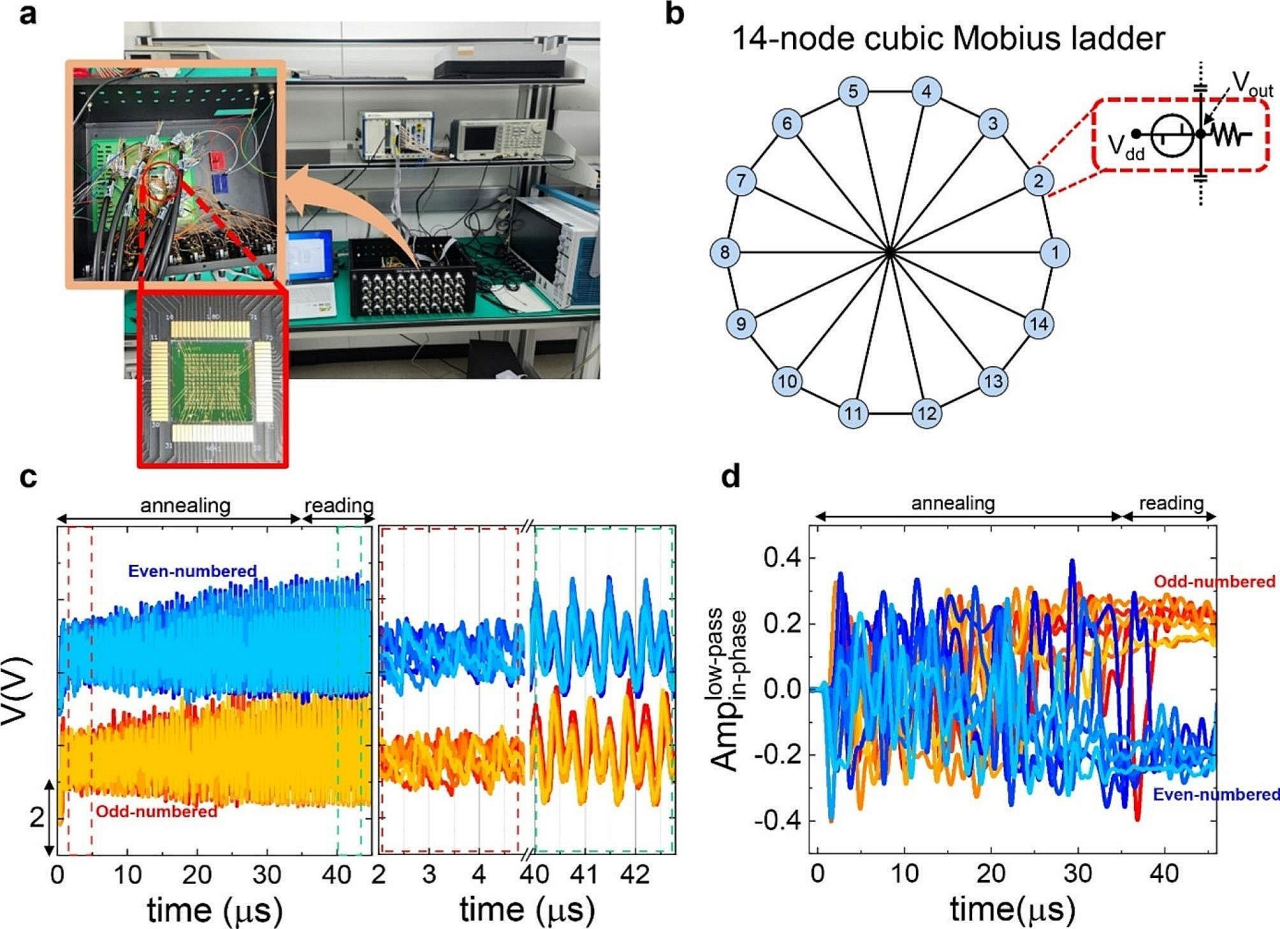
Solving a 14-node MaxCut problem by using coupled OTSNOs. (**a**) A picture of the measurement setup (insets: PCB for configuring connections (orange box) and an array of OTS devices (red box)). (**b**) 14-node MaxCut problem with a Mobius ladder geometry with cubic connections. (**c**) The output waveforms of the 14 oscillators, which are divided into two groups (even-numbered and odd-numbered ones). The waveforms of the even-numbered oscillators are vertically shifted from the odd-numbered oscillators by 3 V for clarity. The right panel shows expansions of the regions before synchronization (red dashed box) and after the synchronization (green dashed box), the latter of which clearly shows the AP relationship between the two groups. (**d**) Temporal evolution of the in-phase component (Amp_in_) of the output waveforms (see the main text)

Based on these results, we have built an IM using OTSNOs and coupling capacitors (*C*_c_=100 pF) as shown in Fig. 3a and, as a benchmark test, have tried to solve a MaxCut problem with a Mobius ladder geometry composed of 14 nodes and cubic connections (see Fig. 3b). We have also tried another geometry, which is presented in Fig. S3 in the Supplementary Information. The details of the measurement are described in the Experimental Section. Representative output waveforms of the 14 OTSNOs are presented in Fig. 3c. We have employed both the second-harmonic injection locking (SHIL) and simulated annealing (SA) techniques [19, 36–38] to improve the reliability of the solution by keeping the system from being stuck to a local minimum. In this technique, all the oscillators are driven by a DC voltage (*V*_dc_) and a modulational AC voltage (*V*_SH_sin(*ω*_SH_*t*)), which locks the oscillation frequency to *ω*_SH_ (= 2*ω*_sync_ ∽ 2.75 MHz, where *ω*_sync_ is the oscillation frequency in the synchronized state). In the right panel, which is the expansion of the waveforms in the range of 40 ∽ 43 µs, it is shown that the odd-numbered OTSNOs oscillate with the nearly same phase while the even-numbered ones also show the synchronized behavior with a clearly distinguished phase.

To quantify the phase difference between oscillators, we have calculated the amplitude of the in-phase component of each *V*_out_(*t*) by applying the low-pass filter (the cut-off frequency of 0.1 MHz) to the product of a reference wave (cos(*ω*_SH_*t*)) and *V*_out_(*t*). Such obtained in-phase amplitude (Amp_in_) of the oscillators are plotted as a function of time in Fig. 3d. It is clearly observed that the oscillators are separated into two groups with the opposite signs of Amp_in_ implying the AP relationship between those two groups of oscillators. In addition, it shows that those two groups are clearly separated 35 µs after applying the bias. Considering that *V*_SH_ gradually increases over 35 µs in the simulated annealing scheme, it indicates that the time-to-solution (*T*_sol_) is expected to be at most 35 µs. From repeated implementations of the same experiment, we have observed *T*_sol_ is in the range of 35 ∽ 40 µs. It might be reduced further by increasing the oscillation frequency. One way is to increase the natural frequency of OTSNOs, which can be achieved by reducing the parasitic capacitance (∽ 100 pF in this work [30]) and the load resistance. Another is to increase the coupling frequency, which can be achieved by using a smaller coupling capacitance in the AFM range in Fig. 2d. Nevertheless, the obtained *T*_sol_ is much shorter than *T*_sol_ ∽ 4 ms reported in a previous work on the hardware implementation of a PTNO-based Ising solver [19] for solving an 8-node MaxCut problem although, in a simulation study, *T*_sol_=30 µs was presented for solving a 100-node MaxCut problem with random cubic connections.

**Fig. 4:**
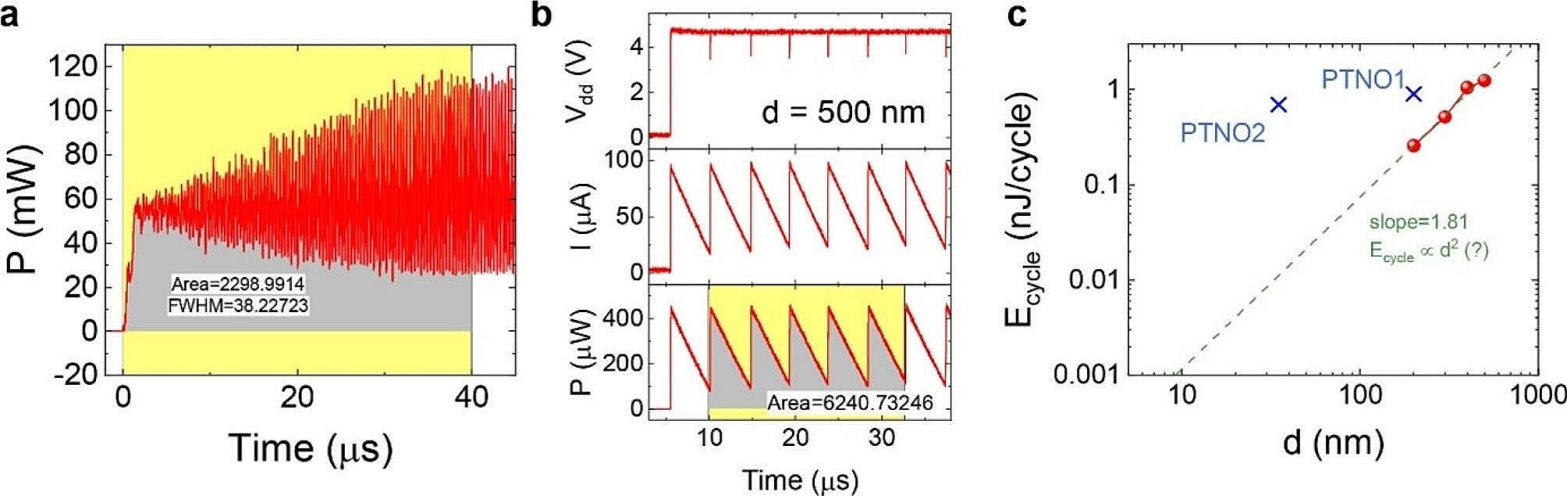
Energy efficiency of the OTSNO-based Ising machine and its scalability. **(a**) Consumed power obtained by *P*(*t*) = *I*(*t*) × *V*(*t*) of all oscillators (see text) as a function of time, resulting in an estimation of the consumed energy (∽ 2.3 µJ) for solving the problem. (**b**) Calculation of the energy consumption per cycle (*E*_cycle_) for an OTSNO using an OTS with the pore size (***d***) of 500 nm. (**c**) Dependence of the *E*_cycle_ of the OTSNO on *d* in comparison with PTNO [19, 39]

The energy-to-solution (*E*_sol_), as another performance metric of the IM, is calculated by integrating the instantaneous power consumption (*P*(*t*) = *V*(*t*)×*I*(*t*), where $V(t)={V_{dc}}+{V_{SH}}\sin (\omega t)$ applied to the system (refer to Fig. S3a) and $I(t)=\sum\limits_{{i=1}}^{{14}} {{I_{i,out}}(t)}$, the sum of the output currents (${I_{i,out}}(t)$) at each node) over *T*_sol_. *P*(*t*) is plotted as a function of time in Fig. 4a, leading to an estimation of *E*_sol_ around 2.3 µJ. Note that this is the lower limit of *E*_sol_ because it does not include the energy consumed in peripheral circuits such as readout circuits. In addition, for large-scale Ising machines, the circuits for controlling the geometry of the problem are needed and consume an amount of energy.

Supposing that the energy consumption in the peripheral circuits does not depend on the types of the oscillator, we have tried to compare the energy consumption in a single OTSNO and PTNO because of the negligible energy consumption in the coupling capacitor (see Fig. S4 in the Supplementary Information) as expected in the pure capacitive circuit. The energy consumption per cycle (*E*_cycle_) is estimated to be ∽ 1.25 nJ/cycle (see Fig. 4b) for a 500 nm (*d*: diameter of the pore)-sized OTSNO and ∽ 0.9 nJ/cycle for a 200 nm (the length of VO_2_ channel)-sized PTNO, respectively. In the OTS device, *E*_cycle_ is expected to be scaled with the dimension of the device because of the shrinkage of the switching volume. Therefore, we have investigated the dependence of *E*_cycle_ of the OTSNO on the diameter of the pore as presented in Fig. 4c (for the waveforms of *V*(*t*), *I*(*t*), and *P*(*t*) corresponding to OTSNOs with various pore sizes, see Fig. S5 in Supplementary Information). For comparison, *E*_cycle_ of the PTNO [19, 39] is also located in the same graph, clearly showing that the OTSNO consumes less energy by about an order than the PTNO with similar device dimensions. The difference is attributed to the difference in the switching mechanisms of the PTNO and the OTSNO. In detail, the PTNO is based on the phase transition of the Mott insulator, which requires heating of a part in the channel material up to the transition temperature. In contrast, the OTSNO is believed to be based on the filling and evacuation of the trap states in the OTS, the electronic process in nature, although there is still controversy about its switching mechanism [24, 26, 34, 40]. In addition, Fig. 4c shows that *E*_cycle_ of the OTSNO scales as *E*_cycle_ ∽ *d*^1.81^ with the diameter of the pore, being close to the expected relation of *E*_cycle_ ∽ *d*^2^ for the case of the uniform current density in the switching material. Therefore, it seems to imply that the reduction in *E*_cycle_ is attributed to the shrinkage of the switching volume inside the cylinder. As a result, *E*_cycle_ of the OTSNO extrapolates to ∽ 1 pJ/cycle at *d* = 10 nm, which enables the development of a highly efficient large-scale IM.

**Fig. 5:**
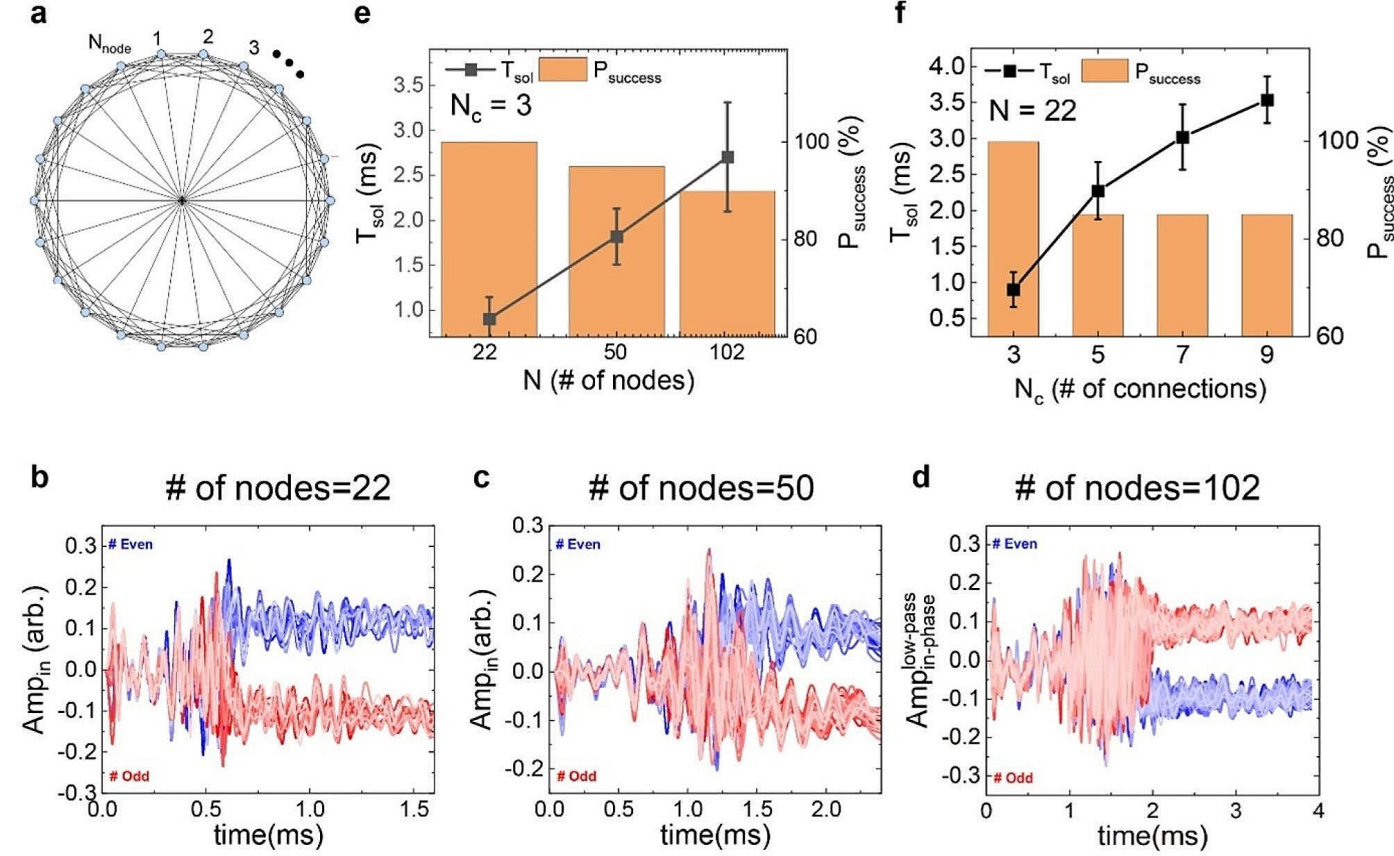
Scalability of the OTSNO-based IM (simulation). (**a**) A Mobius ladder geometry composed of variable numbers of oscillators and connections. (**b**)∽(**d**) Amp_in_ as a function of time with *N* = 22 (**b**), 50 (c), 102 (**d**) with *N*_c_ being fixed at three. (**e**), (**f**) *T*_sol_ (line and symbol, left axis) and *P*_success_ (bar plot, right axis) as a function of the number of nodes (*N*) and the number of connections (*N*_c_) in the Mobius ladder geometry, respectively. In (e) and (f), *N*_c_ and *N* are fixed at 3 and 22, respectively. For each geometry, the simulation was performed 20 times with random initial phases of oscillators, from which the average and the standard deviation are plotted for *T*_sol_

Finally, we have performed a simulation study to investigate the scalability of the OTSNO-based IM. As a typical benchmark task, we have studied the Mobius ladder geometry composed of variable numbers of OTSNOs and connections (see Fig. 5a). Figure 5b ∽ 5d show the representative examples of Amp_in_ of oscillators with varying the number of nodes (*N*) from 22 to 102. The correctness of the obtained solution has been verified by comparing the energy of the solution with that of the ground state. From 20 repetitions of the simulation with varying the initial phases of each oscillator randomly, we have obtained the performance metrics, the success probability (*P*_success_) and *T*_sol_, as shown in Fig. 5e. Note that, as *N* increases, *T*_sol_ is linearly proportional to log(*N*) with a slight decrease in *P*_success_. We have repeated similar simulations with varying the number of connections (*N*_c_) in the way of adding connections to the next nearest neighbors in order with a diagonal connection being fixed. Representative examples of Amp_in_ of oscillators for various geometries are presented in the Supplementary Information (see Fig. S6). In Fig. 5f, it is observed that *T*_sol_ shows a sublinear dependence on *N*_c_ while *P*_success_ decreases slightly with increasing *N*_c_. It implies that, as *N*_c_ increases, local minima are likely to be formed in the energy landscape and the oscillators are trapped in those local minima. Due to the intrinsic chaotic nature of the OTSNO device as mentioned in the discussion of Fig. 2c, we expect that the problem of such local minima might be alleviated in the hardware implementation of the IM using OTSNOs.

## Conclusion

In summary, we have investigated the OTSNO as a candidate nano-oscillator with improved scalability for applications in Ising machines. We have demonstrated that the OTSNO shows robust oscillating behavior with a well-defined frequency, which can be controlled by the gate voltage applied to the FET. We have also demonstrated the synchronization between two coupled OTSNOs with their phase relation (in-phase or anti-phase) controlled by the strength of the coupling elements (electrical resistors or capacitors), which is systematically investigated leading to a conclusion that capacitive coupling provides the larger operation windows with respect to both the value of the coupling capacitance and the device variations of the OTS. Especially for the capacitive coupling, it should be stressed that the anit-phase relationship is observed in a very wide range of the coupling capacitance (2 ∽ 200 pF). It is important to implement Ising solvers for problems with inhomogeneous coupling strengths, for example, the Travelling Salesman Problem (TSP) having coupling strengths that can differ by orders of magnitude.

Finally, we have implemented an Ising machine composed of capacitively-coupled OTSNOs and demonstrated that the solution to a 14-node MaxCut problem can be obtained in 35 µs while consuming no more than 2.3 µJ of energy. Moreover, it is shown that the OTSNO has superior energy scalability with consuming less energy by about an order compared with the PTNO based on the Mott insulator such as VO_2_. We have compared the characteristics of various nano-oscillators in the Supplementary Information (see “2. Supplementary Note: Comparison with other oscillators”). Based on this comparison and considering that the OTSNO provides an oscillator with a minimum size of 6F^2^ with F down to ∽ 14 nm [31, 41], we believe that the OTSNO is highly promising for application to large-scale Ising machines.

## Experimental section

### Fabrication of the OTS

OTS devices are fabricated with a pore-type structure, where the pore size (*d*) is defined by the electron beam lithography in the range of 100 ∽ 500 nm. 60 nm-thick Sb_x_(GeSe)_1−x_ (SGS) layer is used as a switching material because it is found to have a lower threshold voltage (*V*_th_) compared to Ge_50_Se_50_. It is deposited by a co-sputtering technique (magnetron RF sputtering) using Ge, Sb, and GeSe_2_ targets. Pt and TiN are used as the bottom and top electrodes, respectively.

### Characterization of the OTSNO

To configure a frequency-adjustable OTSNO, an OTS device is connected in series to an n-MOSFET device (LND150N3-G, Microchip Inc.). The characteristics of OTS devices and OTSNOs have been investigated using an arbitrary function generator (AFG-3102, Tektronix), an oscilloscope (MSO-58, Tektronix), and a source-measure unit (2635B, Keithley). We have tested several OTSNO devices using OTS devices of various pore sizes and found similar behavior. The results presented in this paper are mainly obtained by using OTS devices with *d* = 500 nm.

### Implementation of the Ising solver

The implementation of the Ising machine composed of OTSNOs including the measurement setup is shown in Fig. 3a and Fig. S3. The OTS devices fabricated on the SiO_2_ substrate are loaded onto a custom-made breakout board. Terminals of the OTS devices are connected to the power supply (*V*_dd_), *R*_L_, and *C*_C_ on a specially designed printed circuit board (PCB). We have used the second-harmonic injection locking (SHIL) technique to lock phases of OTSNO with an oscillation frequency of 2.75 MHz for the injection signal, twice the natural frequency of the single OTSNO. In addition, we have used the simulated annealing technique for the injection signal, whose amplitude gradually increases from 0 V to 2 V over 45 µs. *V*_dc_, *R*_L_, and *C*_C_ are set to 4 V, 1 kΩ, and 100 pF, respectively. An arbitrary function generator (AFG-3102, Tektronix) has been used to bias the driving dc voltage and the SHIL ac voltage. To read the output waveforms of 14 OTSNOs by using an 8-channel oscilloscope (MSO-58, Tektronix), we repeated the measurement runs two times. In the first and second runs, 14 OTSNOs have been read half-and-half relative to oscillator #1.

### SPICE simulation

To perform SPICE simulations, we have used the LTSpice (Analog Device Inc.) software. The OTS device is modeled as a parallel connection between a voltage-controlled switch (VCS) and a parasitic capacitor (100 pF), the former of which is modeled to have hysteretic switching behavior mimicking the OTS with *V*_th_ = 3.3 V, *V*_H_ = 1 V, *R*_on_ = 150 Ω, and *R*_off_ = 10^6^ Ω.

### Electronic supplementary material

Below is the link to the electronic supplementary material.

Supplementary Material 1

Supplementary Material 2

## Data Availability

Not applicable.
