# Supplementary material for: Demonstration of an energy-efficient Ising solver composed of Ovonic threshold switch (OTS)-based nano-oscillators (OTSNOs)
Source: Nano Converg. 2024 May 23;11:20. doi: 10.1186/s40580-024-00429-2 (PMC11116306; doi:10.1186/s40580-024-00429-2)
Supplement: Supplementary file 1 — Supplementary Material 1 [file 40580_2024_429_MOESM1_ESM.pdf]

## Supplementary Information

# Demonstration of an energy-efficient Ising solver composed of Ovonic Threshold Switch (OTS)-based nano-oscillators (OTSNOs)

Young Woong Lee[1,2], Seon Jeong Kim[1], Sangheon Kim[1], Jaewook Kim[1], Jongkil Park[1], YeonJoo Jeong[1], Jaewook Kim[1], Gyu Weon Hwang[1], Seongsik Park[1], Bae Ho Park[2, 3], and Suyoun Lee[1, 4*]

[1]*Center for Neuromorphic Engineering, Korea Institute of Science and Technology, Seoul 02792, Korea*

[2]*Division of Quantum Phases & Devices, Department of Physics, Konkuk University, Seoul 27478, Korea*

[3]*Core Facility Center for Quantum Characterization/Analysis of Two-Dimensional Materials & Heterostructures, Konkuk University, Seoul 05029, Korea*

[4]*Division of Nano & Information Technology, Korea University of Science and Technology, Daejon 34316, Korea*

## S1. Supplementary Note 1: A detailed study on the synchronization behavior of the OTSNO

The synchronization behavior of the OTSNO is investigated by monitoring the response of the OTSNO to an external modulatory AC bias. When synchronized, it is expected that the OTSNO adjusts its oscillation frequency from the natural value to that of the modulatory bias. To this end, an external bias, a mixture of a DC (=4 V) and an AC (=0.23~0.27 V), is applied to the OTSNO, and the output waveform is measured with varying the frequency ($f_{in}$) of the AC component (Fig. S1a). This has been repeated for different gate voltages and typical results obtained with $V_G$=-0.7 V are shown in Fig. S1b. Six representative waveforms are displayed; the bottom two (below synchronization), the middle two (in synchronization), and the top two (above synchronization). Note that the two waveforms in synchronization are clearly distinguishable in that they exhibit a very simple oscillating behavior. In contrast, the other four waveforms show rather complex oscillating behaviors with long-range oscillation envelopes. For quantitative analysis, Fig. S1c shows the FFT of the waveforms in Fig. S1b, which unveils the measured oscillation frequency ($f_{osc}$, triangle) being adjusted to $f_{in}$ (circle) in the synchronized regime while keeping its $f_{nat}$ in the out-of-synchronization regime. In Fig. S1d, the measured oscillation frequency ($f_{osc}$, see Fig. S1c) is plotted as a function of $f_{in}$ at various $V_G$, showing that $f_{osc}$ coincides with $f_{in}$ in a certain range with its center and width depending on $V_G$. In Fig. S1e, the range of $f_{in}$ for the synchronization is presented in the plot of ($f_{in}$-$f_{nat}$) vs. $V_G$, which shows a typical shape called as the "Arnold's tongue". These results indicate that the frequency and the phase of the OTSNO are locked to an external bias, clearly showing synchronization.

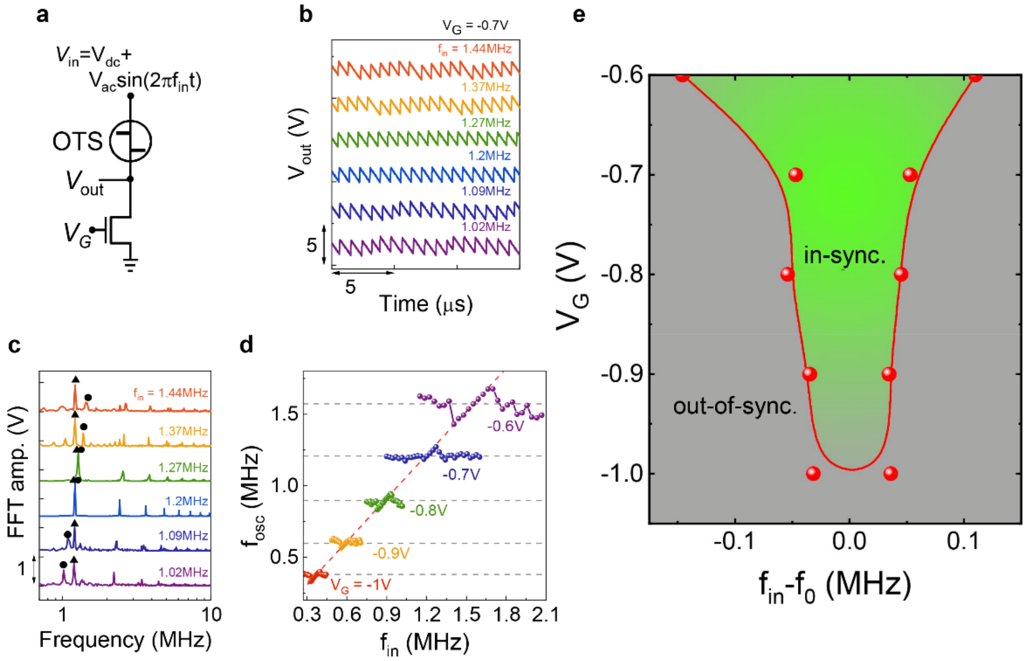


**Fig. S1 Synchronization of OTSNO.** (a) A schematic illustration of the measurement setup, (b) output waveforms of the OTSNO with varying $f_{in}$, where ($V_{dc}$, $V_{ac}$, $V_G$) = (4 V, 0.25 V, -0.7 V), (c) Fast Fourier Transform (FFT) amplitude of the output waveforms, where the circle and the triangle indicate $f_{in}$ and $f_{nat}$, respectively. (d) Oscillation frequency ($f_{osc}$) as a function of $f_{in}$ with varying $V_G$. (e) Range of frequency in synchronization as a function of $V_G$, where $f_{nat}$ is the natural frequency of the oscillator. A solid line is a guide to the eye (B-spline curve).

## S2. SUPPLEMENTARY FIGURES

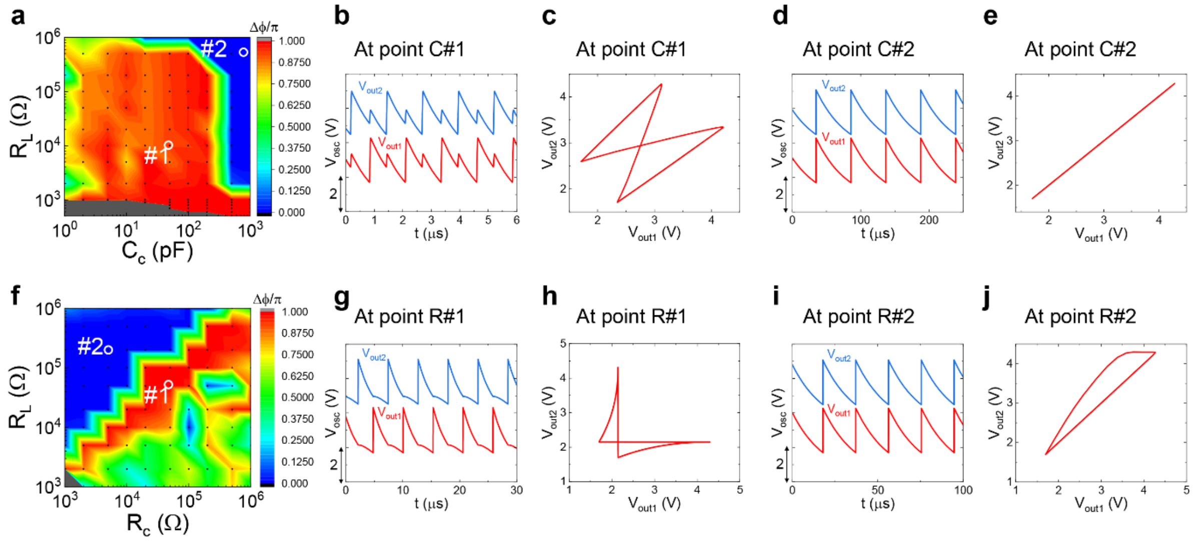


**Fig. S2 Phase difference of two coupled oscillators, coupling-dependent output waveforms and phase portraits.** (a) for two capacitively-coupled OTSNOs, (b)~(e), output waveforms of two oscillators ((b) and (d)) and a phase portrait ((c) and (e)) for the anti-phase coupling case (($R_L$, $C_C$) = (10 kΩ, 50 pF)) and the in-phase coupling case (($R_L$, $C_C$) = (500 kΩ, 500 pF)), respectively. (f)~(j), The same plots for two resistively-coupled OTSNOs, where the anti-phase and in-phase coupling cases correspond to ($R_L$, $R_C$) = (50 kΩ, 50 kΩ) and (20 kΩ, 5 kΩ), respectively.

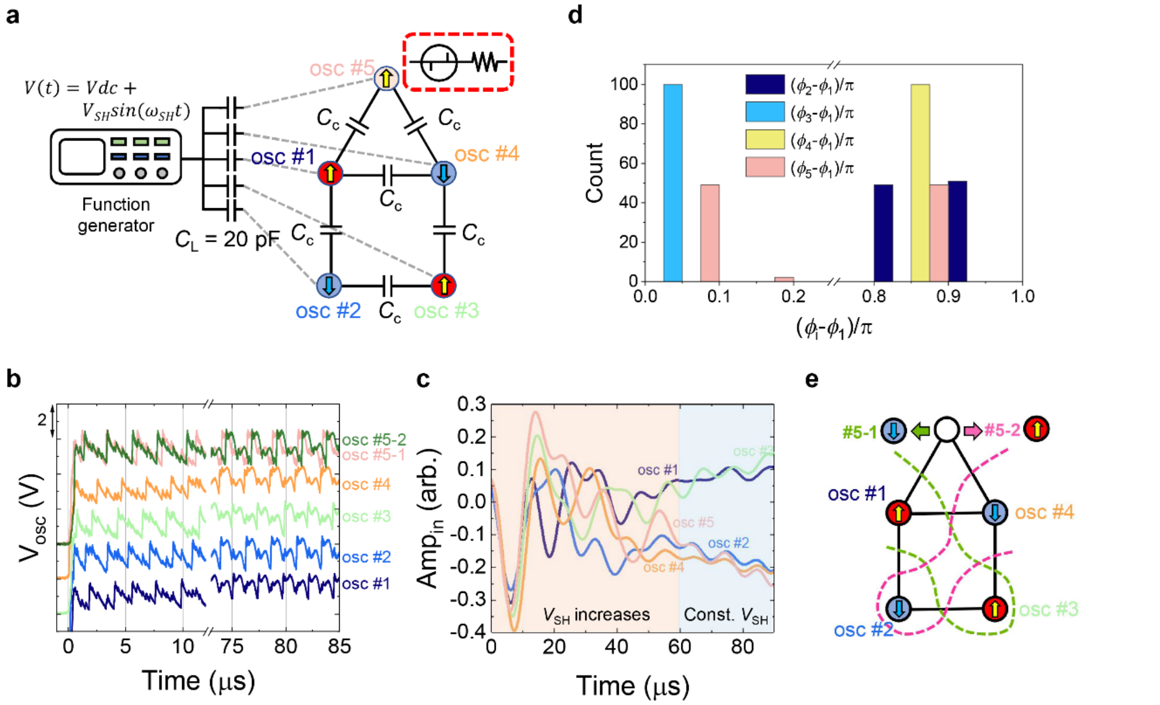


**Fig. S3 Solving a 5-node MaxCut problem by using coupled OTSNOs.** (a) The geometry of an example 5-node MaxCut problem and the setup of an Ising machine. Through the locking capacitor ($C_L$=20 pF), a function generator supplies $V(t) = V_{dc} + V_{SH}\sin(\omega t)$, where the former drives the oscillators and the latter locks the oscillators' frequency at $\omega$ (=2$\omega_{sync}$, where $\omega_{sync}$ is the oscillation frequency of OTSNOs in the synchronized state.). (b) The output waveforms of five oscillators. The waveforms are vertically shifted for clarity. The phase of oscillator #5 is found to be doubly degenerated with the same value as that of oscillator #3 or #4. (c) Temporal evolution of the in-phase component ($Amp_{in}$) of the output waveforms (see the main text). (d) Statistical distribution of the phase difference of the four oscillators ($\phi_i$-$\phi_1$, where i=2, 3, 4, 5) from the reference osc #1, which is obtained by 100 SPICE simulations with varying the initial phase differences. (e), Solution to the five-node MaxCut problem, which is doubly degenerated.

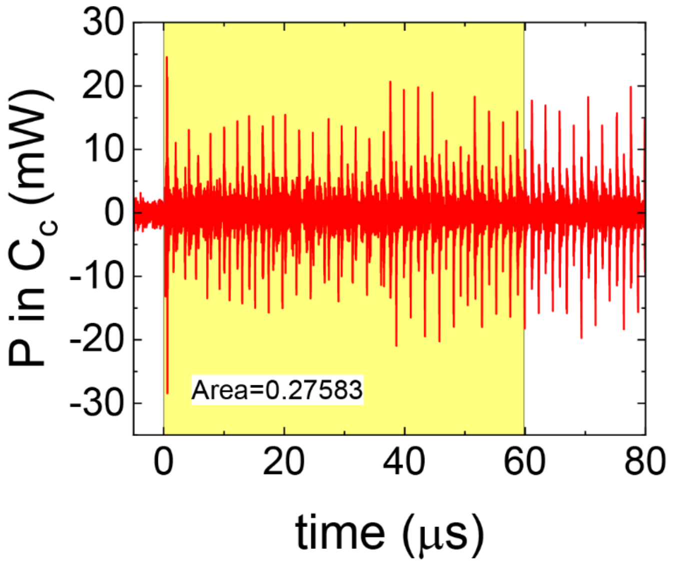


**Figure S4. Power dissipation in the coupling capacitor.**

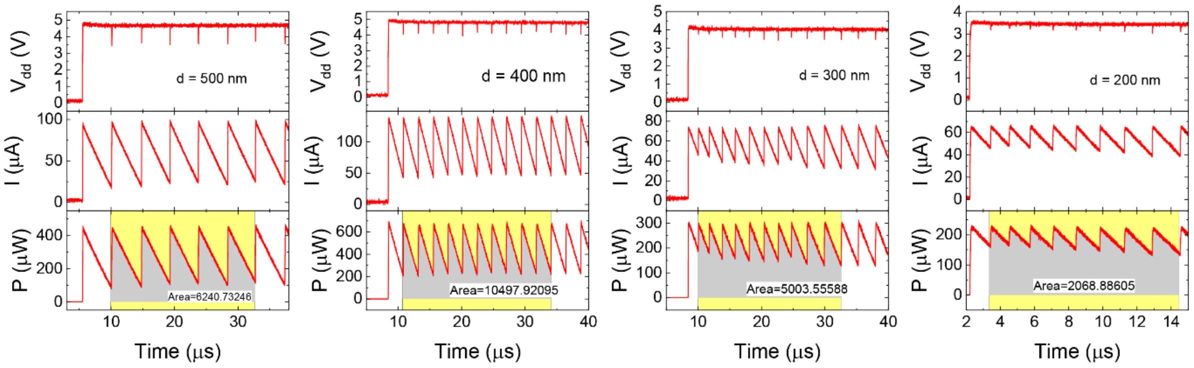


**Fig. S5 Dependence of the oscillating behavior on the pore size (*d*) of the OTS device.**

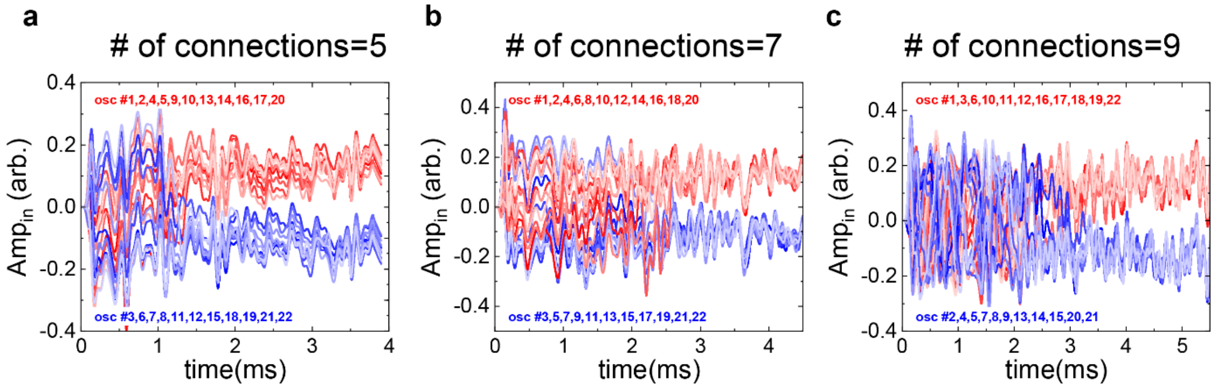


**Fig. S6 Temporal evolution of the in-phase component ($Amp_{in}$) of the output waveforms of oscillators for Mobius ladder geometries with varying the number of connections (simulation).** (a)~(c) $Amp_{in}$ as a function of time with $N_c$=5 (d), 7 (e), 9 (f) with $N$ being fixed at 22.

## S3. SUPPLEMENTARY NOTE: COMPARISON WITH OTHER OSCILLATORS

Electronic oscillators like OTSNO and PTNO are regarded as promising candidates for developing compact and energy-efficient Ising machines. With a working principle different from OTSNO and PTNO, ST (spin-torque)- and SH (spin-Hall)-NO based on MTJ require an external magnetic field, which gives these oscillators a disadvantage in terms of compactness and energy efficiency.

In comparison with PTNO, the OTSNO is advantageous in the energy efficiency and the operation temperature. With respect to energy efficiency, the PTNO has a weakness in its switching mechanism relying on Joule heating for phase transition. In addition, the off-state resistance of the PTNO is around 10 kΩ [see Fig. 8 in M. Jerry *et. al.*, "Ultra-low power probabilistic IMT neurons for stochastic sampling machines." 2017 Symposium on VLSI Technology. IEEE, 2017], lower than the OTSNO by about 1/1000 times. Such a low off-state resistance leads to a large energy consumption due to a high leakage current. With respect to the operation temperature, the upper limit of the operation temperature of the PTNO is set by the metal-insulator transition temperature ($T_{MIT}$), 67 $^{o}$C for $VO_2$. In addition, since the switching in the Mott insulator relies on the Joule heating, the switching characteristics are expected to be sensitive to the ambient temperature. In contrast, in OTS, the upper limit of the operation temperature is set by the crystallization temperature of the switching material, which is higher than 400 $^{o}$C for GeSe in this work. Finally, the ease of process is another advantage of the OTSNO. GeSe in the OTS is deposited by RF sputtering while the PTNO requires quality-controlled $VO_2$ which can be obtained through more sophisticated deposition techniques like molecular beam epitaxy (MBE) at an elevated growth temperature.

We summarized the above comparison in the following Table S1.

| | **LC-oscillator** | **Ring oscillator** | **Spin-torque (or Spin-Hall) oscillator** | **PTNO ($VO_2$, $NbO_2$, …)** | **OTSNO (This work)** |
|---|---|---|---|---|---|
| **Scalability (in size)** | Not good | Not good | Good | Good | Good |
| **Energy efficiency** | Low | Decent | N/A (expected to be low due to external magnetic field) | Decent | Good |
| **Leakage current (standby power)** | Bad | Good | - | Decent (off state ~ $10^4$ Ω) | Good (off state ~ $10^7$ Ω) |
| **Operation temp.** | - | - | $T < T_{Curie}$ of the ferromagnet in MTJ | $T < T_{MIT}$ of the Mott insulator (~ 67 °C for $VO_2$) | $T < T_{crys}$ of the chalcogenide in the OTS (> 400 °C for GeSe) |
| **Fabrication process** | Simple | Decent | Complex | Complex (MBE and/or growth at high temp.) | Simple (Sputtering at room temp.) |

**Table S1. Comparison between electronic oscillator devices**
