# Supplementary material for: Demonstration of an energy-efficient Ising solver composed of Ovonic threshold switch (OTS)-based nano-oscillators (OTSNOs)
Source: Nano Converg. 2024 May 23;11:20. doi: 10.1186/s40580-024-00429-2 (PMC11116306; doi:10.1186/s40580-024-00429-2)
Supplement: Supplementary file 2 — Supplementary Material 2 [file 40580_2024_429_MOESM2_ESM.pdf]

Response to reviewers' comments

Manuscript ID: NCON-D-24-00032

Title: Demonstration of an energy-efficient Ising solver composed of Ovonic Threshold Switch (OTS)-based nano-oscillators (OTSNOs)

Authors: Young Woong Lee; Seon Jeong Kim; Jaewook Kim; Sangheon Kim; Jongkil Park; YeonJoo Jeong; Jaewook Kim; Gyu WeonHwang; Seongsik Park; Bae Ho Park; Suyoun Lee

* Corresponding author, E-mail: slee_eels@kist.re.kr

We thank the reviewers for their valuable comments. We have revised our manuscript based on the reviewers' constructive suggestions. The revised manuscript has become scientifically more rigorous with improved readability, and hence, we believe that it meets the standards for publication in the *Nano Convergence.* Below we address the comments in detail.

Reply to Reviewer #1

Reviewer's comment:

This study experimentally demonstrated an Ising solver based on ovonic threshold switch-based nano oscillators for unconventional computing. By comparing the two coupling elements, the authors revealed that it is advantageous to use capacitor to obtain a larger operating window. The manuscript was well organized, but minor comments need to be addressed before publication.

Reply: We thank the reviewer for his/her positive evaluation of our manuscript and valuable comments which help us to improve the quality of the manuscript. We respond to each specific comment below.

1) Because synchronization of independent oscillations plays an important role in solving COP, the injection locking process that clearly shows pre- (or post-) synchronization should be included.

Reply: We thank the reviewer for this valuable comment. Conforming to the comment, we have revised our manuscript and a figure (Fig. 3c, and we present it here below (Fig. R1)) to

include the waveforms before and after the synchronization while solving the 14-node MaxCut problem. It clearly shows that the oscillators are little synchronized under the SHIL of low amplitude whereas they are synchronized under the SHIL of high amplitude.

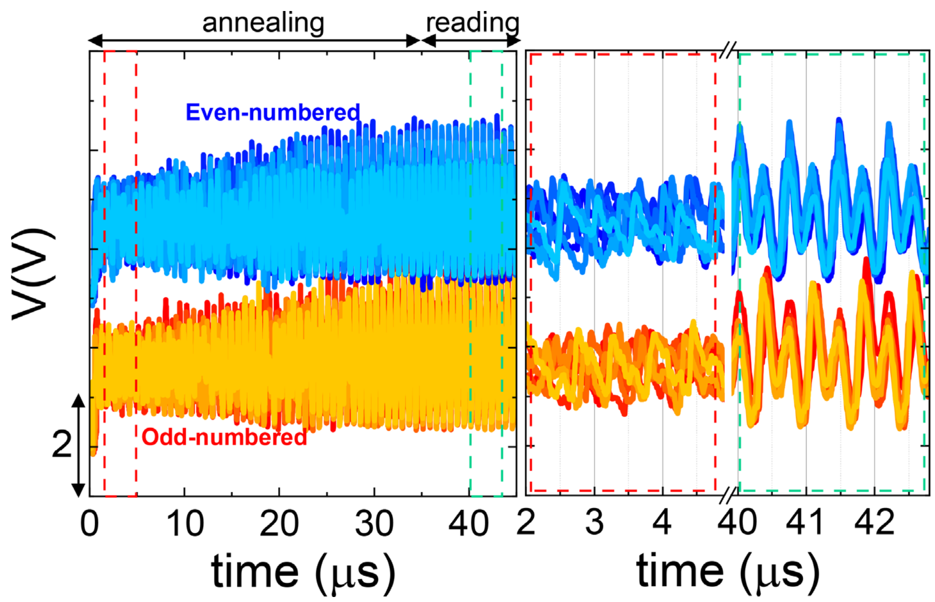

Fig. R1 The output waveforms of the 14 oscillators, which are divided into two groups (even-numbered and odd-numbered ones). The waveforms of the even-numbered oscillators are vertically shifted from the odd-numbered oscillators by 3 V for clarity. The right panel shows expansions of the regions before synchronization (red dashed box) and after the synchronization (green dashed box), the latter of which clearly shows the AP relationship between the two groups.

List of Changes (highlighted yellow in manuscript):

- Page 17: Fig. 3c and the caption

2) In the current version of the manuscript, it is difficult to identify the configuration of the oscillators (e.g., fully connected). It should be explained in detail how the 14 oscillators are constructed and how the output signals on the chip are measured.

Reply: We thank the reviewer for this careful comment. The geometry of the 14-node MaxCut problem in this work is in Fig. 3(b). It is called as the Mobius ladder with cubic connections, where each oscillator is connected to three other oscillators, two nearest neighbors, and the

other diagonal one. In our experiment, we designed a printed circuit board (PCB, Fig. R2(a)) which contains a coupon having a lot of OTS devices and jumper pin sockets for configuring the connections. A schematic diagram of the configuration is shown below (Fig. R2(b)). The output signals were measured at the output nodes in Fig. R2(b) using a multi-channel oscilloscope.

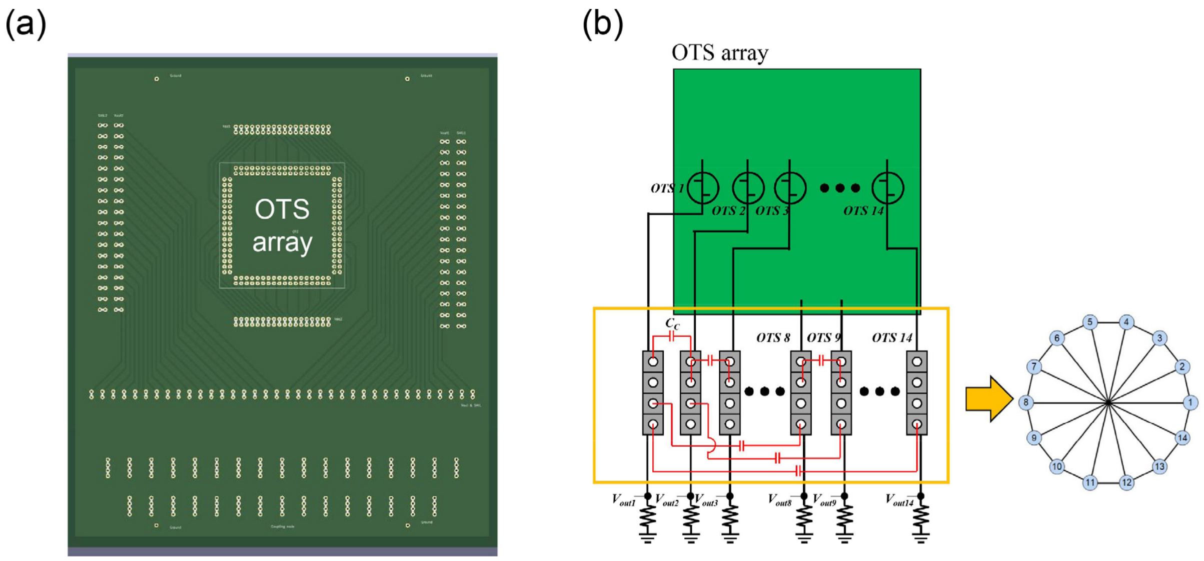

Fig. R2 (a) Printed circuit board (PCB) for building an IM based on OTSNO, (b) a schematic diagram showing the connection between OTSNOs.

3) It would be good to include additional discussion or outlook to further accelerate the time, when oscillations are split into two distinct amplitudes. This perspective can provide insight to readers.

Reply: We thank the reviewer for this valuable comment. Conforming to the comment, we have revised our manuscript to include additional discussion to reduce the time-to-solution as follows.

List of Changes (highlighted yellow in manuscript):

- Page 8, lines 8-12: "… in the range of 35 ~ 40 μs. It might be reduced further by increasing the oscillation frequency. One way is to increase the natural frequency of OTSNOs, which can

be achieved by reducing the parasitic capacitance (~ 100 pF in this work) and the load resistance. Another is to increase the coupling frequency, which can be achieved by using a smaller coupling capacitance in the AFM range in Fig. 2d."

Reply to Reviewer #2

Reviewer's comment:

The authors propose and experimentally demonstrate an Ising machine (IM) based on Ovonic Threshold Switch(OTS) nano-oscillators. The authors characterized OTSNO synchronization properties for a second harmonic injection locking and two types of coupling - capacitive and resistive. OTSNOs have much higher frequency as compared to Phase-Transition nano-oscillators (PTNO) and, consequently, allow for much better time-to-solution and energy efficiency parameters in the design of Ising machines.

The OTSNO-based IM was benchmarked experimentally with a simple MaxCut with a Mobius ladder geometry composed of 14 nodes and cubic connections. The authors have also performed a simulation study to investigate the scalability of the OTSNO-based IM.

The authors have also extrapolated energy consumption per cycle E_cycle to 1 pJ/cycle if the diameter of OTS was reduced to10 nm which gives a prospect for highly energy-efficient IMs of middle scale.

Overall, it is an interesting and timely paper that will be of interest to a broad readership. Here are some suggestions to improve the novelty and relevancy of their work with literature and to benchmark the OSTNO-based IMs using more general combinatorial problems:

Reply: We deeply thank the reviewer for his/her thorough review of our manuscript and appreciate that he/she recommends the manuscript for publication.

1) In the introduction, when the authors mentioned a large-scale optical parametric oscillator (OPO)-based coherent Ising machine, I would suggest adding a reference to the latest realization of CIM that already has 100,000 spins (https://doi.org/10.1126/sciadv.abh0952).

Reply: We thank the reviewer for this valuable comment. Conforming to the comment, we have revised our manuscript to include the reference (Reference #8).

2) The authors could have mentioned another spintronic realization of Ising machine that has a similar to their realization power consumption but faster time-to-solution parameter and better scalability potential due to its time-multiplexing architecture - a spinwave Ising machine (SWIM) (https://doi.org/10.1038/s42005-023-01348-0).

Reply: We thank the reviewer for this valuable comment. Conforming to the comment, we have revised our manuscript to include the reference to the spinwave-based Ising machine.

List of Changes (highlighted yellow in manuscript):
- Page 2, line 26 - Page 3, lines 1: "… instead of using optical wave, spinwave- and surface acoustic wave-based IMs (SWIM[9] and SAWIM[10], respectively) …"

3) The authors mentioned that the OPO-based CIMs have a drawback in their scalability because they use long fiber that require a lot of space. However, CIMs are in fact the largest IMs in physical implementation and by their principle of operation intrinsically have better scalability than IMs based on physical oscillator arrays where the number of physical interconnections for dense matrices for just a 2000-spin problem would already exceed a million which represent a significant problem for an analog circuit meant to be manufactured with 2.5D mass production technology. Moreover, even a 20-km-long optical fiber used in the latest 100,000-spin CIM was assembled as a coil consuming no more than a few cubic decimeters. The main problems leading to large space consumption are: 1. bulky optical components such as a parametric phase-sensitive amplifier. 2. the need for optical tables which arises due to increased sensitivity to vibrations; 3. poor thermal stability due to extremely large phase accumulation in the fiber caused by the combination of its length and short light wavelength. These problems were recently solved in a time-multiplexed surface acoustic wave-based Ising machine (SAWIM). (https://doi.org/10.48550/arXiv.2311.06830).

Having said that, I would like to point out that another fundamental problem of all time-multiplexed IMs is the reduced interaction time between oscillators, as most of the time they

spend in the delay line unchanged. IMs based on physical oscillator arrays interact continuously, allowing for intrinsically much faster time-to-solution parameters, taking into account the scaling factor of oscillation frequency. As consequence of fast time-to-solution parameter the power consumption is also expected to be low. However, physical oscillator array IMs seem to be significantly more limited by the maximum number of spins. Therefore, I would recommend to change the reasoning of why it is important to develop IMs based on physical oscillator arrays despite the existence of CIMs, SWIMs and SAWIMs.

Reply: We thank the reviewer greatly for this careful and valuable comment. We have learned a lot from this comment. Conforming to the comment, we have revised our manuscript to include references about SWIMs and SAWIMs and to point out the problem of those techniques. In addition, we have changed the reasoning of why it is important to develop IMs based on physical oscillator arrays.

List of Changes (highlighted yellow in manuscript):

- Page 2, line 24 - Page 3, lines 5: "…a lot of space, bulky optical components such as a parametric phase-sensitive amplifier, and optical tables to minimize the perturbations by vibrations. To resolve these problems, instead of using optical wave, spinwave- and surface acoustic wave-based IMs (SWIM[9] and SAWIM[10], respectively) were introduced very recently. These techniques, however, rely on the time-multiplexing technique for the coupling between oscillators, where the coupling strength is calculated in an FPGA (field-programmable gate array) and imposed to oscillators sequentially, spending most of the time in the delay line. It possibly poses a problem of the time-to-solution and the energy-to-solution increasing with the number of oscillators."

4) The Moebius ladder graphs are frequently used in many initial experiments having small number of spins, but also known to be too simple to really demonstrate the ability to solve the hard instances of NP hard optimization problems (see https://arxiv.org/abs/2008.00466). I would suggest to use for benchmarking randomly generated matrixes and/or instances used from BiqMac library (https://biqmac.aau.at/biqmaclib.html) or generated using Rinaldi's graph generator (Giovanni Rinaldi. Rudy. http://www.user.tu-chemnitz.de/~helmberg/rudy.tar.gz, 1998.).

Reply: We thank the reviewer for this careful comment. We agree with the reviewer's opinion that the demonstrated task (solving the 14-node MaxCut problem) in this work is too simple to verify the ability of the proposed IM to solve the hard instances of NP hard optimization problems. Nevertheless, the main purpose of this work is to introduce a new oscillator device based on Ovonic threshold switch, which can improve the speed and the energy efficiency of Ising machines in comparison with those based on the other electrical oscillators. Since the feasibility of application to Ising machines was tested in the MaxCut problem with Mobius geometry in most of the previous studies using the other types of oscillator, we feel that it is fair to compare our proposed device with the others keeping the task similar. Nevertheless, we feel that it is an interesting topic to challenge to solve the MaxCut problem with geometries generated from the BiqMac library or Rinaldi's graph generator. We hope that we can report the study in our future work.

*5) The authors define time-to-solution as the time it takes to OTSNO-based IM to freeze in a final state including the annealing time of 35us. However, for already medium-scale machines the usual way to define time-to-solution parameter includes taking into account the success probability as defined in (https://doi.org/10.1007/978-981-99-5072-0):TTS_99=tau_single_run*log(1-0.99)/log(1-p_success) where tau_single_run is the time that it takes to obtain a single solution and p_success is the probability to obtain 99%-accurate solution. This measure is especially relevant when an IM is benchmarked with a standard set of MAX-CUT problems as Biq Mac.*

Reply: We thank the reviewer for this careful comment. We agree with the reviewer's opinion that the definition of the time-to-solution in this work is different from the conventional definition. However, achieving 99 % success probability implies that the measurement should have been performed at least 100 times. We feel that it is a tedious job for such a small-sized problem in this work. As mentioned in our manuscript, we performed the test several times and obtained the solution in 35-40 us with 100 % probability, verifying the reproducibility of our system. Therefore, we believe that the time-to-solution for the definition of the 99 % success probability will not be much different from that range.

6) In the simulation study devoted to the demonstration of the scalability of the OTSNO-based IMs I would also expect BiqMac lib for more general benchmarking.

Reply: We thank the reviewer for this careful comment. As mentioned in the response to comment #3, we feel that it is fair to compare our device with other oscillator devices with the same task in the scalability aspect, too. We will try to solve the problems in our future work including the study on scalability.

7) I believe that the paper would benefit from including a discussion on how combinatorial problems with coefficients that can differ in magnitude by several orders (for example, when mapping Travelling Salesman Problems on Ising machines) can be mapped on OTSNO-based IMs. How much one can vary the capacitance of the coupling capacitor and still observe antiferromagnetic coupling between spins. Perhaps, it can be deduced from Fig.2.

Reply: We thank the reviewer for this valuable comment. Indeed, as the next step in this work, we are currently trying to solve such a problem, specifically the "number partitioning" problem. The problem is to divide a group of numbers into two subgroups so that the sum of each subgroup is the closest to each other. In the transforming of the problem to an Ising machine, each number in the group is mapped to an oscillator and the coupling strength should be set proportional to the product of two numbers corresponding to oscillators. Then, the solution is obtained by the groups of oscillators with its phase of 0 or π. In a preliminary test, we tried a six-node number partitioning problem and successfully obtained the solution. In that experiment, the coupling capacitance is varied in the range of 1 ~ 200 pF. We are now trying to solve the problem on a little larger scale and will report the result as soon as possible. Conforming to the reviewer's comment, we have revised our manuscript to include a discussion about Ising solvers for such kinds of problems.

List of Changes (highlighted yellow in manuscript):

- Page 11, lines 3 - 7:

" Especially for the capacitive coupling, it should be stressed that the anit-phase relationship is observed in a very wide range of the coupling capacitance (2 ~ 200 pF). It is important to implement Ising solvers for problems with inhomogeneous coupling strengths, for example,

the Travelling Salesman Problem (TSP) having coupling strengths that can differ by orders of magnitude."

8) When assessing energy efficiency, the authors took into account the power consumption of OTSNO and coupling capacitors. However, for large-scale arbitrary problems OTSNO-based IM will also require the circuits to control the coupling capacitance, i.e. vary it or completely switch off the coupling. I believe a comment in this regard would benefit the paper.

Reply: We thank the reviewer for this careful comment. As the reviewer pointed out, the energy consumed during solving a problem includes not only the energy in the oscillator devices and coupling elements, but also that in the peripheral circuits for driving and reading oscillators. In addition, for a large-scale Ising machine, the circuits for controlling the geometry of the problem are needed and consume an amount of energy. Conforming to the reviewer's comment, we have revised our manuscript to include that point.

List of Changes (highlighted yellow in manuscript):

- Page 8, lines 21 - 24:

" Note that this is the lower limit of Esol because it does not include the energy consumed in peripheral circuits such as readout circuits. In addition, for large-scale Ising machines, the circuits for controlling the geometry of the problem are needed and consume an amount of energy."

Minor comments) (1) The y-axis scale is not clear in Fig.3c.

Reply: We thank the reviewer for this comment. Conforming to the comment, we have revised the caption of Fig. 3c to be more clear. In addition, note that Fig. 3c is revised to include the waveforms before synchronization conforming to the comment from another reviewer.

List of Changes (highlighted yellow in manuscript):

- Page 17, lines 7 – 8 (Figure caption of Fig. 3)

" The waveforms of the even-numbered oscillators are vertically shifted from the odd-numbered oscillators by 3 V for clarity."

Reply to Reviewer #3

Reviewer's comment:

This manuscript presents a nano-oscillator comprising an ovonic threshold switch and resistors to construct an energy-efficient Ising machine. The authors demonstrate a robust and well-defined oscillating feature controllable by an external gate voltage applied to an FET. Additionally, they illustrate that the fabricated device, when coupled with a capacitor, exhibits a larger operational window and less variation compared to those coupled with resistors. The study showcases that the 14-node MaxCut problem can be solved faster with lower energy consumption using the suggested device platform, in contrast to a conventional phase-transition nano-oscillator-based Ising machine. The introduction and the strategy for addressing a combinational optimization problem are intriguing, suggesting potential for extension to a large-scale hardware system given the simplicity of device and circuit structures. Here, I offer several comments aimed at enhancing the quality of the manuscript.

Reply: We thank the reviewer for his/her positive evaluation of our manuscript and valuable comments which help us to improve the quality of the manuscript. We respond to each specific comment below:

1) In page 5, the author stated that the oscillation frequency can be controlled by the V_G owing to discharging period over R_{FET}. Would you elaborate on this statement? Why does the fnat of OTSNO increase as the VG increases?

Reply: We thank the reviewer for this careful comment. The operation principle of the OTSNO is based on the voltage dividing between the OTS (characterized by R_{on} and R_{off}) and the load resistor (R_{load}), which depends on the resistance of the OTS under the condition of $R_{on} << R_{load} << R_{off}$. In detail, when the OTS is in its OFF state, the applied voltage drops mostly across the OTS, turning the OTS on. Subsequently, when the OTS is in its ON state, the applied voltage now mostly drops across the load, turning the OTS off. This procedure completes one cycle of oscillation of the OTSNO.

In addition, the OTS device is modeled as a voltage-controlled switch with a parallel-connected parasitic capacitor (C_p). Therefore, the alternating transition of the OTS between its

ON and OFF states takes time proportional to RC delay. When the OTS is in its OFF state, C_p is charged up with the characteristic time of $(R_{load}+R_{off})C_p$, which corresponds to the descending phase of V_{out} of the oscillation. In contrast, when the OTS is ON, C_p is discharged with the characteristic time of $(R_{load}+R_{on})C_p$, which corresponds to the ascending phase of the oscillation. Since R_{off} is much larger than R_{on}, the descending phase takes the longer time than the ascending phase. For this reason, fnat depends on the R_{load}, which can be controlled by the gate voltage of the FET acting as the load resistor. By the way, the "discharging period" in our original manuscript was mistaken and we have revised our manuscript to fix the error.

List of Changes (highlighted yellow in manuscript):

- Page 4, line 4

"… charging and discharging period …"

2) Would you define the read voltage for R_{off} and R_{on}? Could you plot the log-scale of I-V switching curve in Fig. 1c and clarify the ON-OFF ratio?

Reply: We thank the reviewer for this comment. R_{off} was around 2 ~ 5 MΩ, being measured by applying a dc bias of 0.2 V much below the holding voltage (~ 1 V). R_{on} was obtained from the slope of the characteristic current-voltage curve. From the slope in Fig. 1c (Fig. R3a for convenience), the dynamic resistance is estimated to be ~ 560 Ω with a R_{load} = 300 Ω connected in-series, leading to R_{on} ~ 260 Ω. Therefore, the ON-OFF ratio was ~ 10^4 in our OTS device. In Fig. R3b, the log-scale of *I-V* switching curve is plotted. It was measured by a pulsed *I-V* measurement in order to avoid permanent damage to the device. In this measurement, a triangular voltage pulse is biased and the current was read from the voltage drop across the load resistor using an oscilloscope, which has the voltage resolution much poorer than a dc voltmeter or a dc current meter. Therefore, the OFF-state trace in Fig. R3b is very noisy. For this reason, R_{off} was measured by applying a dc bias.

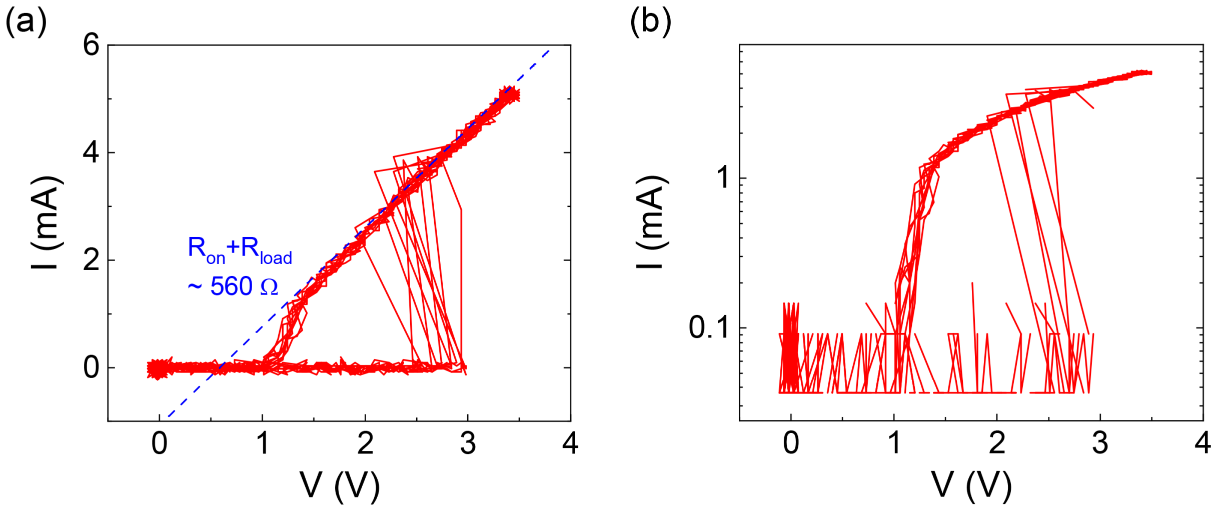

Fig. R3 (a) Characteristic switching *I-V* curve in the linear-linear scale. A blue dashed line represents a linear fitting curve to the *I-V* curve for the ON-state trace, giving an estimation of R_{on} of ~ 200 Ω. (b) The same curve in log-linear scale.

3) Revise a typing error ("Tlhe" in page 8) and caption (f0 fnat) of x-axis in Fig. S1e.

Reply: We thank the reviewer for this careful comment. We have revised our manuscript to fix the errors.

4) For Fig. 2, why does the tolerance of the coupled oscillator matter for Ising machine?

Reply: We thank the reviewer for this careful comment. The tolerance to the device variations means the range of device variations, under which the phase relationship is preserved. If the tolerance is too small, the Ising machine is vulnerable to unavoidable device variations. Therefore, the observation of the tolerance of up to ± 20 % of the variation of V_{th} implies the robustness of the Ising machine based on the OTSNO.

5) Fig. 3 shows the picture of measurement system. However, it seems difficult to grasp the connections and device scheme with this picture.

Reply: We thank the reviewer for this careful comment. The geometry of the 14-node MaxCut problem in this work is in Fig. 3(b). It is called as the Mobius ladder with cubic connections,

where each oscillator is connected to three other oscillators, two nearest neighbors, and the other diagonal one. In our experiment, we designed a printed circuit board (PCB, Fig. R4(a)) which contains a coupon having a lot of OTS devices and jumper pin sockets for configuring the connections. A schematic diagram of the configuration is shown below (Fig. R4(b)). The output signals were measured at the output nodes in Fig. R4(b) using a multi-channel oscilloscope.

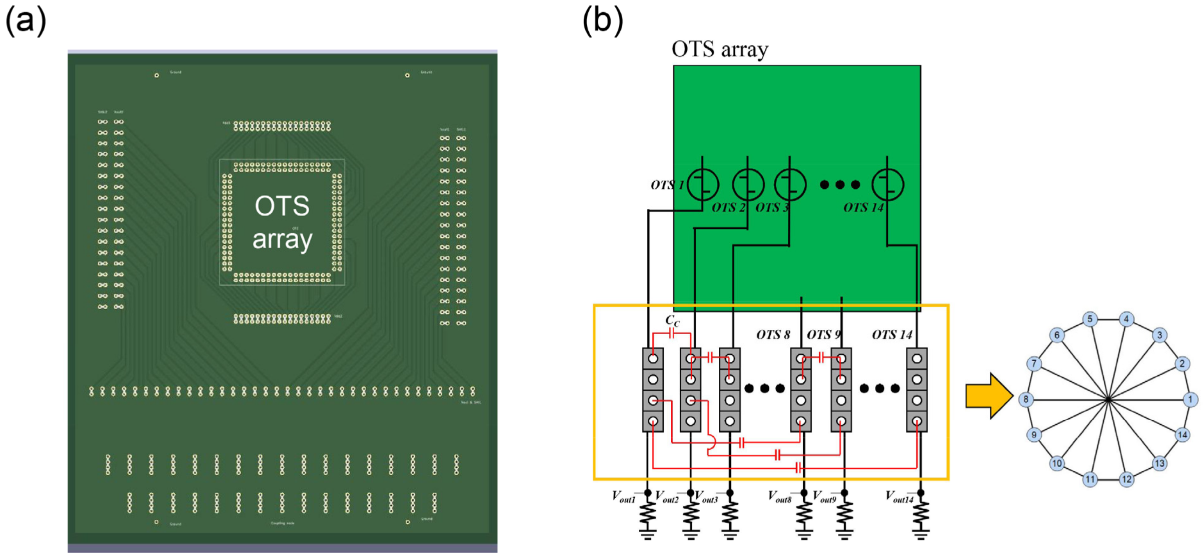

Fig. R4 (a) Printed circuit board (PCB) for building an IM based on OTSNO, (b) a schematic diagram showing the connection between OTSNOs.

6) It needs to be consistent significant figures shown in Figures. Especially, the inset numbers in Fig. 4 have different valid numbers.

Reply: We thank the reviewer for this careful comment. After we scrutinize Fig. 4, we think that the reviewer might be confused about the unit. In Fig. 4a, the energy consumption is estimated from the area under the power curve, which is ~ 2300 (mW•μs=nJ) = 2.3 μJ. In Fig. 4b, the energy consumption per cycle (E_{cycle}) is estimated from the area under the power curve (~ 6240 μW•μs = 6.24 nJ) divided by the number of cycles (five in Fig. 4b), resulting in E_{cycle} ~ 1.25 nJ/cycle. This figure is consistent with the point in Fig. 4c.
